# Integrated weighted gene co‐expression network analysis reveals biomarkers associated with prognosis of high‐grade serous ovarian cancer

**DOI:** 10.1002/jcla.24165

**Published:** 2022-01-08

**Authors:** Bo Wang, Shan Chao, Bo Guo

**Affiliations:** ^1^ Maternal & Child Health Research Institute, Shenzhen Baoan Women's and Children's Hospital Jinan University Shenzhen China; ^2^ Institutes for Shanghai Pudong Decoding Life Shanghai China

**Keywords:** biomarker, high‐grade serous ovarian cancer, hub gene, proteomics, WGCNA

## Abstract

**Background:**

Ovarian cancer is the gynecologic tumor with the highest fatality rate, and high‐grade serous ovarian cancer (HGSOC) is the most common and malignant type of ovarian cancer. One important reason for the poor prognosis of HGSOC is the lack of effective diagnostic and prognostic biomarkers. New biomarkers are necessary for the improvement of treatment strategies and to ensure appropriate healthcare decisions.

**Methods:**

To construct the co‐expression network of HGSOC samples, we applied weighted gene co‐expression network analysis (WGCNA) to assess the proteomic data obtained from the Clinical Proteomic Tumor Analysis Consortium (CPTAC), and module‐trait relationship was then analyzed and plotted in a heatmap to choose key module associated with HGSOC. Subsequently, hub genes with high connectivity in key module were identified by Cytoscape software. Furthermore, the biomarkers were selected through survival analysis, followed by evaluation using the relative operating characteristic (ROC) analysis.

**Results:**

A total of 9 modules were identified by WGCNA, and module‐trait analysis revealed that the brown module was significantly associated with HGSOC (cor = 0.7). Ten hub genes with the highest connectivity were selected by protein‐protein interaction analysis. After survival and ROC analysis, ALB, APOB and SERPINA1 were suggested to be the biomarkers, and their protein levels were positively correlated with HGSOC prognosis.

**Conclusion:**

We conducted the first gene co‐expression analysis using proteomic data from HGSOC samples, and found that ALB, APOB and SERPINA1 had prognostic value, which might be applied for the treatment of HGSOC in the future.

## Graphical abstract

We applied weighted gene co‐expression network analysis (WGCNA) to assess the proteomic data obtained from the Clinical Proteomic Tumor Analysis Consortium (CPTAC). A total of 9 modules were identified by WGCNA, and module‐trait analysis revealed that the brown module (cor = 0.7) was significantly associated with HGSOC. Ten hub genes with the highest connectivity were selected by protein‐protein interaction analysis. After survival and ROC analysis, ALB, APOB and SERPINA1 are suggested to be the biomarkers of HGSOC prognosis.

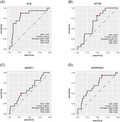

## 1 INTRODUCTION

Ovarian cancer is the gynecologic cancer with the highest mortality in the world, and ranks third in the incidence of gynecologic cancers, next to cervical cancer and endometrial cancer; 313,959 new cases and 207,252 deaths were estimated to have happened in 2020.^1^ High‐grade serous ovarian cancer (HGSOC), belonging to epithelial ovarian cancer (EOC), is the most common and fatal type of ovarian cancer, and the first leading cause of ovarian cancer‐related deaths.^2^, ^3^, ^4^ Since the ovaries are located deeply in the pelvic cavity, and there are no specific symptoms and effective screening methods of the early stages of HGSOC, most patients are often in an advanced clinical stage (stage III/IV) with metastasis at the time of diagnosis. The standard treatment of HGSOC is similar to other ovarian cancers, which is to perform primary debulking surgeries followed by chemotherapy combining platinum and paclitaxel.^2^ But 80% of patients with advanced HGSOC will experience recurrence and chemotherapy resistance, leading to a 5‐year survival rate of 30%,^5^ and more and more evidences indicate that the metastasis‐prone characteristics of HGSOC play an important role in its relatively poor prognosis.^6^, ^7^ In advanced HGSOC, tumor cells have spread out from the ovaries and pelvic organs to the peritoneum and abdominal organs, which could impede the normal function of vital organs in the abdomen by uncontrolled proliferation, as well as function of circulatory and respiratory system by generating large amounts of ascites and increasing intra‐abdominal pressure,^5^, ^8^ which eventually causing the HGSOC patients to die of various complications. However, patients diagnosed with early‐stage HGSOC usually have a good prognosis after standard clinical interventions. Therefore, the studies on the biomarkers for early diagnosis or judging the prognosis of HGSOC are of great help to develop new effective therapies and improve the prognosis of patients with HGSOC.

Over the past two decades, with the breakthrough of high‐throughput sequencing technology, a huge amount of next‐generation sequencing data of various human tissues has been accumulated, which has greatly promoted the progress of biomedicine research, such as the screening of probable cancer prognostic biomarkers. Specifically, in the field of ovarian cancer research, a variety of mRNA‐ or lncRNA‐based signatures have been identified for survival prediction in patients with ovarian cancer. For example, one study analyzed the differentially expressed genes in samples of ovarian cancer at different clinical stages and discovered specific gene co‐expression modules related to the clinical stage and finally identified COL3A1, COL1A1, COL1A2, KRAS and NRAS as potential prognostic genes for ovarian cancer.^9^ Another study showed that 5 lncRNAs (LINC00664, LINC00667, LINC01139, LINC01419 and LOC286437) could be used as independent risk factors for recurrence of ovarian cancer.^10^ Meanwhile, other researchers have proved that changes in expression levels of genes were related to platinum resistance in ovarian cancer.^11^ But almost all of these integrated analyses are performed at the transcriptome level, not at the proteome level. It is well known that protein is the main executor of life activities, and all life activities depend on the correct function of protein. And with the advancement of research methods for proteomics and the accumulation of public proteomic data, performing integrated proteomics analysis is completely feasible at present.

In the post‐genomic era, the consensus that the occurrence of complex diseases such as cancer is not determined by a single gene that has gradually gained the approval of most researchers. Gene co‐expression network research can provide us with information about gene expression correlations and potential functional relationships, thereby assisting us comprehend biological systems and explore the relationship between the relevant functional genes.^12^ And weighted gene co‐expression network analysis (WGCNA) is a method for concretizing this idea, which has been comprehensively applied to multiple cancer‐associated studies to identify hub genes related to various traits.

Recent proteomics studies using ovarian tissue samples from HGSOC patients by Clinical Proteomic Tumor Analysis Consortium (CPTAC) have aided us better understand the mechanism of tumorigenesis from a novel insight and have also identified some candidate therapeutic targets.^2^, ^13^, ^14^ In this study, we applied WGCNA to reanalyze these published proteomic data in order to discover proteins and pathway related to occurrence and development of HGSOC and identified a significant correlation between the brown module and the HGSOC, clinical stage, histological grade and patient survival time. Ten hub genes were selected from this module and verified by survival and relative operating characteristic (ROC) analysis. Finally, it was identified that ALB, APOB and SERPINA1 might be the potential biomarkers related to the prognosis of HGSOC. To our knowledge, this is the first study of prognostic biomarkers for HGSOC applying proteomic data, which provides some new insights into the occurrence and progress of HGSOC.

## 2 MATERIALS AND METHODS

### 2.1 Proteomic data collection and pre‐processing

The quantitative proteomic data and clinical information of HGSOC were obtained from CPTAC Data Portal (https://cptac‐data‐portal.georgetown.edu/studies/filters/primary_site:Ovary). And the unshared peptides expression matrices analyzed through the Common Data Analysis Pipeline were used for subsequent data analysis. Samples lacking information about clinical stage, tumor histological grade and survival time and proteins with missing value of relative abundance among all samples were excluded from our study. Other sample inclusion criteria and data processing procedures were described in previous studies,^2^, ^13^, ^14^ in short, that were (1) five samples were removed causing without *TP53* mutation; (2) the median values of relative protein abundance over all proteins in every sample were calculated and re‐centered to value of 0; (3) the normalized relative protein abundances of overlapping samples were averaged and used as their protein abundances. Finally, 2,892 proteins were identified to perform WGCNA analysis.

### 2.2 Co‐expression network construction and module detection

We used the WGCNA package (Version 1.70, https://CRAN.R‐project.org/package=WGCNA) to construct the co‐expression network for the identified proteins.^15^ First, sample cluster analysis was carried out by the function hclust of WGCNA package to assess whether there were any significant outliers in the selected sample. Next, a suitable soft threshold power for scale‐free network construction was calculated and chosen with the function pickSoftThreshold of WGCNA package. After, an adjacency matrix was built to bring about weighted separation of co‐expression with the chosen soft threshold power value.^16^ Co‐expression similarity for paired proteins from adjacency matrix was calculated by measuring the topological overlap dissimilarity, and then we got a topological overlap matrix (TOM) for next identification and similarity analysis of co‐expression gene modules and combination of similar modules with the following major parameters: deepSplit of 2, minModuleSize of 15 and mergeCutHeight of 0.3. The resulting protein co‐expression network was visualized as the heatmap based on dissimilarity of TOM with hierarchical clustering dendrogram, and the number of proteins in each module was counted and plotted with the barplot (ggplot2, Version 3.3.5, https://CRAN.R‐project.org/package=ggplot2).

### 2.3 Identification of module‐trait correlations and module preservation

The correlations between modules and clinical traits including sample type, clinical stage, pathological grade and survival time were assessed by the Pearson correlation coefficients and a heatmap was plotted to demonstrate the correlation value of interaction between modules and traits. The student *t*‐test was used to get the *p* value of the correlation, and a *p* value of < 0.05 was considered statistically significant. The brown module with the highest value of correlation coefficients was mainly focused on and the correlation between gene significance (GS) for HGSOC and module membership (MM) in brown module was checked to identify module‐trait associations.

### 2.4 Functional annotation of modules

Gene ontology (GO) enrichment analysis and KEGG pathway enrichment analysis were performed for brown module via Cluster Profiler package (Version 3.16.1, https://bioconductor.org/packages/release/bioc/html/clusterProfiler.html),^17^ and the top 5 results with adjusted *p* value of < 0.05 of enrichment analysis was visualized using chord diagram by GOplot package (Version 1.0.2, https://CRAN.R‐project.org/package=GOplot).^18^

### 2.5 Identification of hub genes

Genes closely connected to the intramodular nodes are regarded as hub genes which usually have more important biological function than other nodes.^19^ Protein‐protein interaction (PPI) network analysis was performed via the online database Search Tool for Retrieval of Interacting Genes (STRING, Version 11, https://string‐db.org/),^20^ then the result of PPI analysis was imported to Cytoscape software (Version 3.8.0) to screen out top 10 hub genes ranked by degrees in the network of key modules using CytoHubba plug‐in (Version 0.1).^21^, ^22^

### 2.6 Kaplan‐Meier survival and ROC analysis

Survival analysis of hub genes was conducted and visualized via the Survival package (Version 3.2–11, https://CRAN.R‐project.org/package=survival) and Survminer package (Version 0.4.9, https://cran.r‐project.org/package=survminer), respectively. The relative protein abundances and overall survival time from our data were used to plot the Kaplan‐Meier curves. The cut‐off values of the hub genes to separate the samples were determined by Survminer package. The hazard ratio (HR) was calculated with 95% confidence interval. Log‐rank tests were performed to provide the statistical significance, and *p* value of < 0.05 was considered statistically significant. To evaluate the possibility of the hub genes acting as the biomarkers, we conducted the receiver operating characteristic (ROC) analysis and the ROC curve was plotted by ggplot2 package. And the area under the ROC curve (AUC) was calculated by the pROC package.^23^

### 2.7 Gene set enrichment analysis

Gene set enrichment analysis (GSEA) of the biomarkers was performed with GSEA software (Version 4.1.0).^24^ The package “h.all.v7.4.symbols.gmt”of the Molecular Signature Database (MsigDB, https://www.gsea‐msigdb.org/gsea/msigdb/) was selected as reference gene set.^25^ The normalized enrichment scores and *p* value were generated, and *p* value of < 0.05 was considered statistically significant.

## 3 RESULTS

### 3.1 Identification of co‐expression modules using WGCNA

**FIGURE 1:**
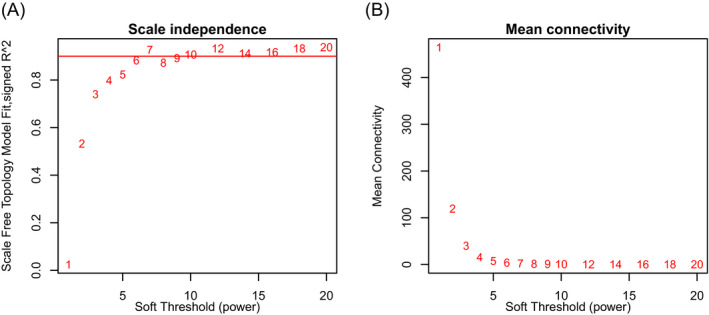
Scale independence and mean connectivity of co‐expression modules based on different soft threshold

**FIGURE 2:**
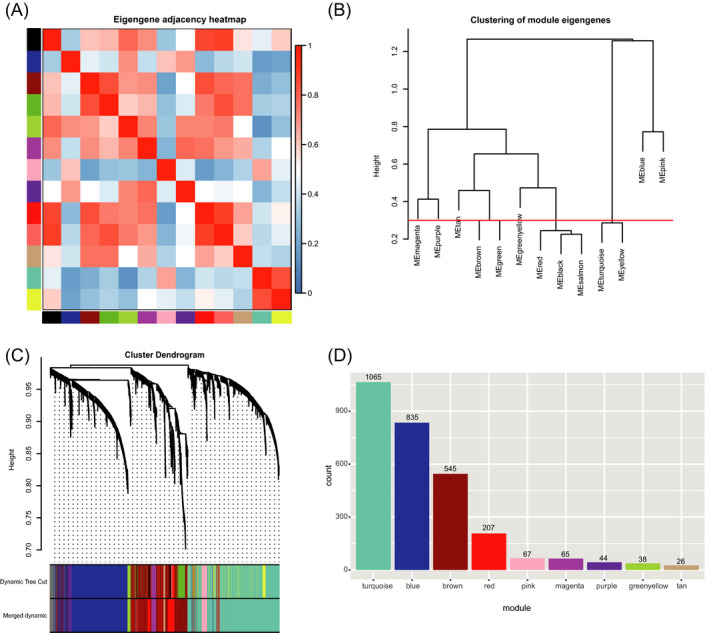
Identification of co‐expression modules using WGCNA. (A) Eigengene adjacency analysis of different modules plotted with a heatmap. Red represents a high correlation, and blue represents a low correlation. (B) Hierarchical cluster analysis of different modules. The red line represents cut height of 0.3. (C) Hierarchical clustering of genes with dissimilarity based on topological overlap is shown with the modules detected and the merged modules. (D) Count of gene in different modules

**FIGURE 3:**
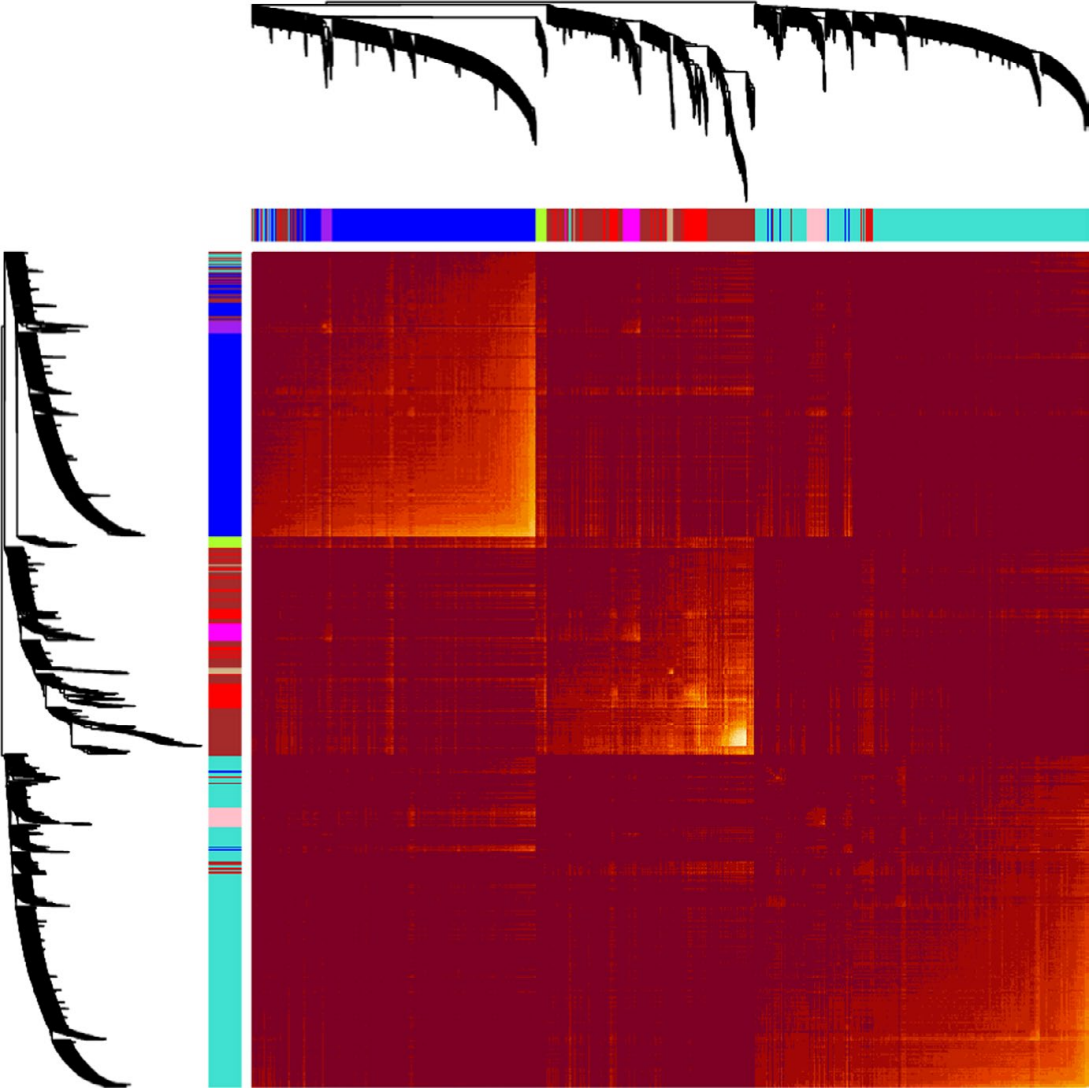
Interaction analysis of co‐expression genesin different modules. The different colors of the horizontal and vertical axes represent different modules. The yellow brightness in the middle indicates the degree of correlation between different modules based on the topological overlap matrix (TOM)

It is believed that genes with comparable co‐expression patterns are usually controlled by relative regulatory manner or have similar or parallel pathways of functional interaction.^12^ In this study, we obtained the proteomic data from the CPTAC database according to Data Use Agreement. After the necessary quality control and manual check and screening, a matrix of relative abundance of 2,892 proteins from 25 normal fallopian tube and 235 HGSOC samples with clinical information were selected to construct the co‐expression networks. To ensure the reliability of the co‐expression network, sample clustering analysis was performed to investigate the outliers among all samples, and no outliers were detected (Additional file 1). Finally, the relative abundances of these 2,892 proteins and 260 samples were applied to identify the modules of co‐expression genes (Additional file 2). To obtain scale‐free topology, a value of 7 of soft threshold power was selected based on scale independence analysis (R^2 = 0.927), and the mean connectivity analysis was also relatively high under this soft threshold power (Figure 1). Thirteen modules were generated firstly, and 4 modules were merged into adjacent modules due to their high relevance of module eigengenes with adjacent modules, thus a total of 9 modules were included in our subsequent analysis (Figure 2A, B). The hierarchical clustering dendrogram of proteins also showed the analogous results (Figure 2C). Numbers of proteins in each module were displayed in Figure 2D, and the detailed result is summarized in Additional file 3. Afterward, the interactive relations among all modules and all proteins were visualized by a heatmap plot based on TOM (Figure 3).

### 3.2 Brown module significantly relates to HGSOC

**FIGURE 4:**
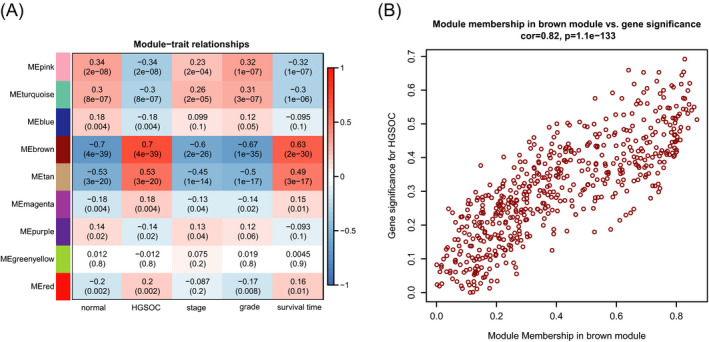
Module‐traitrelationship analysis. (A) The relationship between different modules and trait including sample type, stage, grade and survival time was visualized with a heatmap. Red represents a positive correlation and blue represents a negative correlation. (B) The correlation of gene significance for HGSOC versus the module membership in the brown module is depicted as a scatter plot. The correlation coefficient is calculated through Pearson's correlation analysis

To determine whether co‐expression modules were associated with sample types and clinical traits, the module‐trait relationship analyses were performed (Figure 4A). The brown module was identified to be positively related to HGSOC with the top relevance and to survival time and negatively to stage and grade. In addition to brown module, the tan module also showed a lower correlation with above traits compared with brown module. Then, the correlation between GS for HGSOC and MM in brown module was analyzed, and the correlation coefficients value was of 0.82 (Figure 4B).

### 3.3 Functional enrichment analysis of proteins in brown module

**FIGURE 5:**
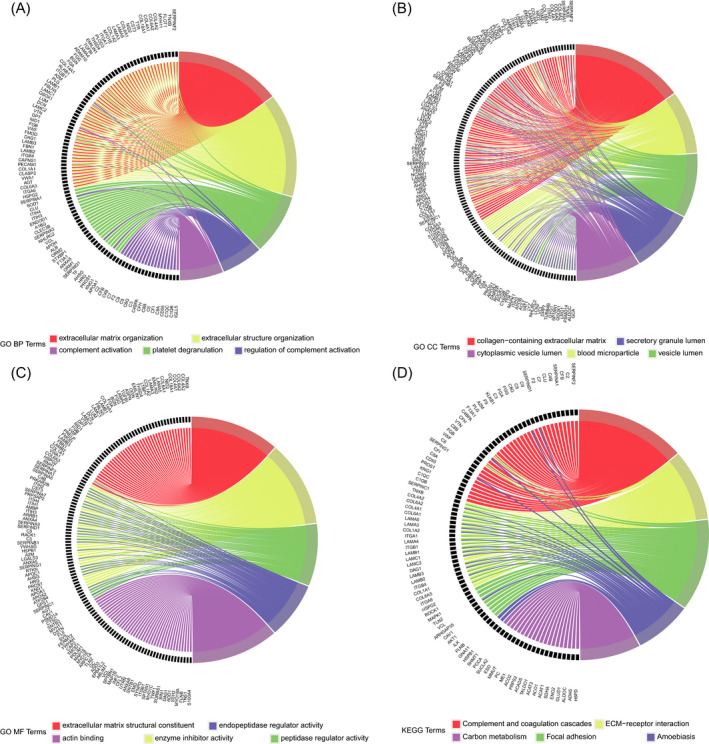
Enrichment analysis of proteins in brown module with the chord diagram. (A) Biological process category of GO enrichment analysis. (B) Cellular component category of GO enrichment analysis. (C) Molecular function category of GO enrichment analysis. (D) KEGG pathway enrichment analysis

To further explore the biological function of the proteins in brown module, GO and KEGG pathway enrichment analyses were performed (Figure 5 and additional file 4). The top 5 terms ranked by adjusted *p* value of results of enrichment analysis were visualized with the chord diagram. The proteins in brown module were significantly enriched in terms of extracellular matrix organization, extracellular structure organization, platelet degranulation, regulation of complement activation and complement activation of BP category (Figure 5A), and collagen‐containing extracellular matrix, blood microparticle, vesicle lumen, secretory granule lumen and cytoplasmic vesicle lumen of CC category (Figure 5B), and extracellular matrix structural constituent, enzyme inhibitor activity, peptidase regulator activity, endopeptidase regulator activity and actin binding of MF category (Figure 5C). While for the enrichment of KEGG pathway, these proteins were mainly enriched in complement and coagulation cascades, ECM‐receptor interaction, focal adhesion, amoebiasis and carbon metabolism (Figure 5D).

### 3.4 Identification of biomarkers related to the prognosis of HGSOC

**FIGURE 6:**
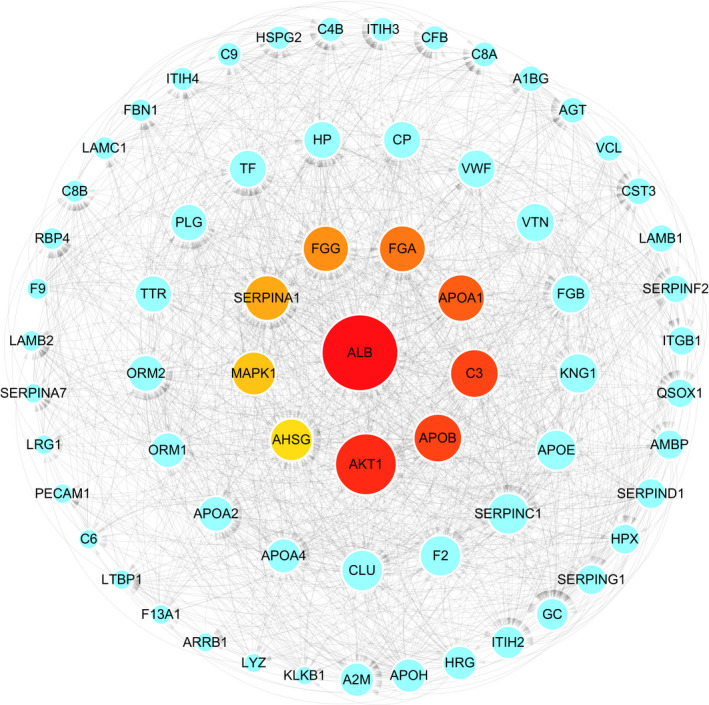
Diagram of interaction network of the proteins in brown module. The large red node is the node with a high degree of connectivity, while the small blue node is the node with a low degree of connectivity

**FIGURE 7:**
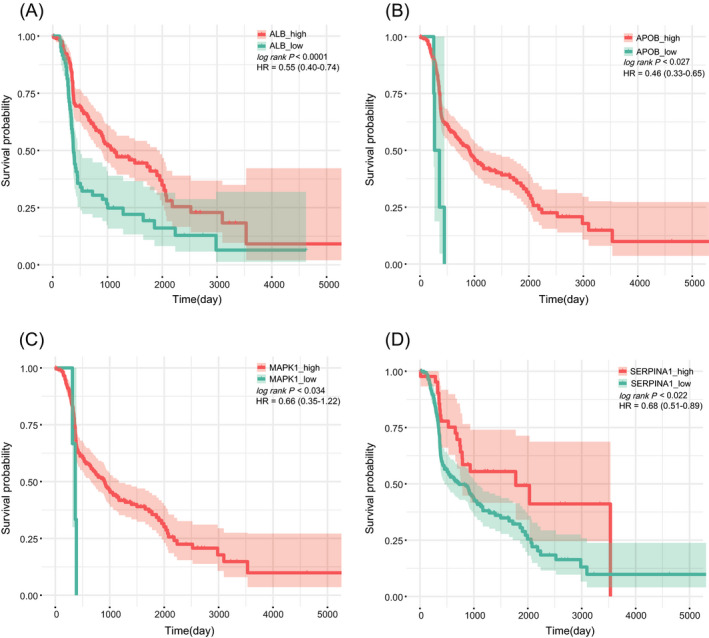
Survival analysis. The survival analysis of ALB (A) APOB (B) MAPK1 (C) and SERPINA1 (D) based on our proteomic data and patient information

**FIGURE 8:**
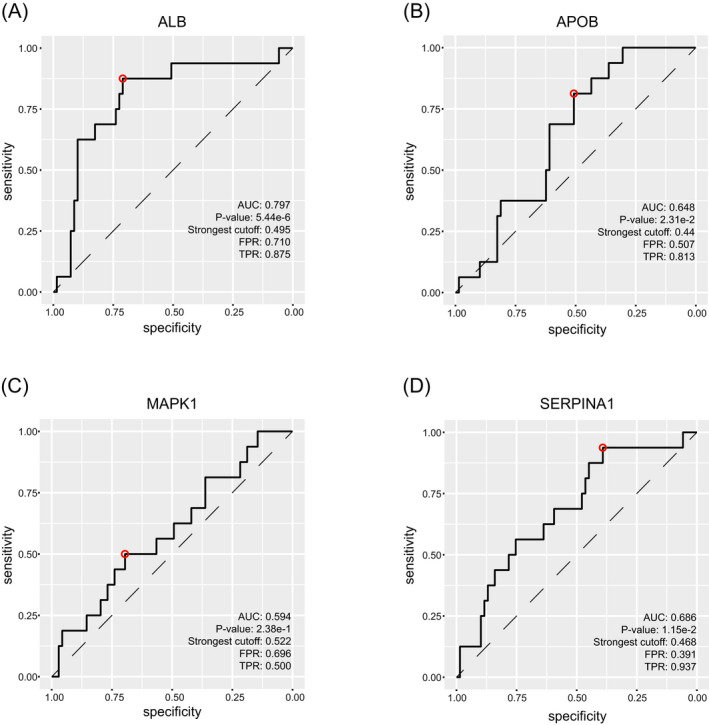
ROC analysis. (A) ALB. (B) APOB. (C) MAPK1. (D) SERPINA1. The red circle represents the optimal threshold of ROC

The 545 proteins in brown module were uploaded and analyzed by STRING database to identify the interaction between them, and then the top 10 proteins (ALB, AKT1, APOB, C3, APOA1, FGA, FGG, SERPINA1, MAPK1 and AHSG) ranked by degree of connectivity were selected as hub genes by cytoHubba plug‐in of Cytoscape software. The network with neighbors generated by topological analysis of degree is shown in Figure 6. Furthermore, the relationships between the 10 hub genes and the overall survival time of patients with HGSOC were analyzed based on our data. Notably, low expression of ALB, APOB, MAPK1 and SERPINA1 significantly correlated with the poor survival time of patients with HGSOC (*p* < 0.05; Figure 7). Next, the ROC analysis revealed that ALB (AUC: 0.797), APOB (AUC: 0.648) and SERPINA1 (AUC: 0.686) had high diagnostic value and could serve as biomarkers for the prognosis of HGSOC (Figure 8).

### 3.5 Gene set enrichment analysis

**FIGURE 9:**
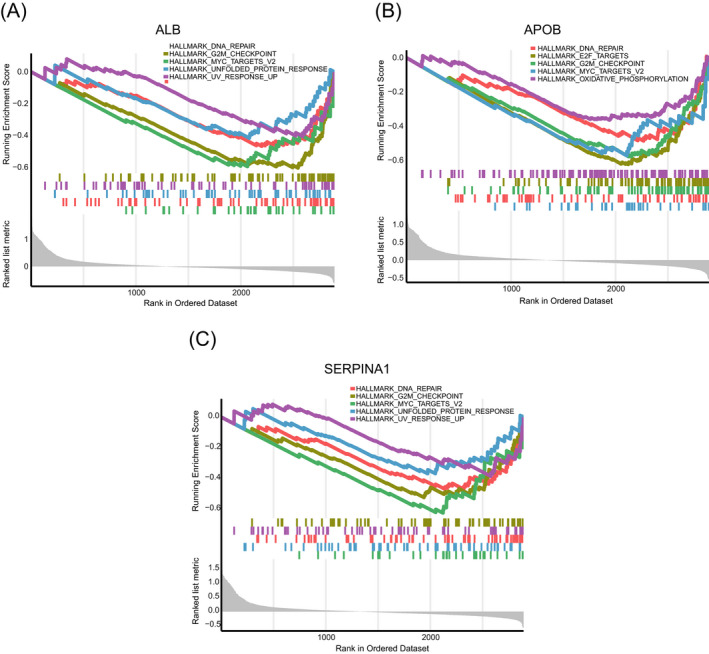
Gene set enrichment analysis. The top 5 enriched entries of low‐expression group of ALB (A) APOB (B) and SERPINA1 (C)

To further understand the biological function of ALB, APOB and SERPINA1 in HGSOC, we performed the GSEA based on our proteomic data. As shown in Figure 9, all of low expression of ALB, APOB and SERPINA1 were significantly associated with terms of “DNA repair,” “G2 M checkpoint” and “MYC targets V2.”

## 4 DISCUSSION

High‐grade serous ovarian cancer remains the most common type of ovarian cancer with the highest incidence and the strongest fatality rate all over the world, and there is no definite research conclusion on its tumorigenesis mechanism. Meanwhile, due to the lack of effective early screening methods, most patients with HGSOC are diagnosed at the advanced stage, accompanied by extensive peritoneal metastasis, and furthermore, most patients will experience tumor recurrence, the above two factors together lead to a very poor prognosis for patients with HGSOC.^2^, ^5^ However, the 5‐year survival rate of patients with early stages of HGSOC is as high as 92%, which is 62% higher than that with later stages of HGSOC,^26^ which suggests the possibility that patients with HGSOC can benefit from efficient early screening methods. Many researchers have conducted extensive research in this field and have also discovered some novel diagnostic markers with clinical application value.^9^, ^10^, ^11^ But the objects of these studies are almost at the transcript level, and protein, as a more direct manifestation of the life activities of cells, organs and even the body, may be a better research target for the screening of high‐efficiency diagnostic or prognostic biomarkers. More importantly, current researches show transcript levels that by themselves are not sufficient to predict protein levels in many scenarios.^27^ Furthermore, with the accumulation of proteomic of data in public databases, it also provides more feasibility to do this kind of research.

In this study, we obtained proteomic data of HGSOC samples from the CPTAC database. Then, these data were assessed by WGCNA, and the brown module was identified to be significantly related to HGSOC. Interestingly, the brown module is not only significantly related to HGSOC but also significantly related to the patient's survival time and significantly negatively related to the clinical stage and histological grade of HGSOC. The results of enrichment analysis of proteins in brown module show that most of these proteins are related to the organization and function of the extracellular matrix (ECM) components including collagen (COL1A1, COL1A2, COL4A1, COL4A2, COL6A1, COL6A2, etc.), proteoglycan (LUM, DCN), laminin (LAMA4, LAMB1, LAMB2, LAMC1) and other proteins as linkers to connect the above proteins (NID1, PRELP,TNXB), covering almost all types of ECM components, and most of these proteins were downregulated in our data and were consistent with the results of previous proteomics studies.^2^ The metastasis‐prone characteristics of HGSOC play an important role in its relatively poor prognosis.^6^, ^7^ Tumor invasion and metastasis is a complicated pathological process which involving interactions between tumor cells and various biologically active molecules from tumor microenvironment including ECM,^28^ and for malignant tumor cells derived from epithelial cells like HGSOC, epithelial‐mesenchymal transition (EMT) is the key first biological step for these tumors cells to metastasize, which is accompanied by disorders of ECM composition and organization, and in turn enhancing tumor cell mobility and protecting tumor cells from immune attack via collagen remodeling, and finally promoting the invasion and metastasis of HGSOC.^5^, ^29^, ^30^ These facts not only prove the credibility of the correlation between the brown module and the clinical stage, histological grade and survival time of patients but also indirectly prove the validity of the results of our analysis.

Moreover, the PPI analysis was conducted to screen hub genes, which were further verified with survival and ROC analysis. Finally, ALB, APOB and SERPINA1 showed significant correlations with the patient's prognosis, and moreover the AUCs of ALB, APOB and SERPINA1, especially ALB were high enough to serve as the biomarkers for the prognosis of HGSOC. ALB encodes the secreted and main protein of human blood, lymph, cerebrospinal and interstitial fluid, which plays important roles in a variety of physiological functions.^31^ And ALB has been reported to participate in the development and treatment of tumors with different mechanisms. First, previous studies have shown that ALB is significantly inhibited during cancer‐related systemic inflammation, which is regulated by a variety of cytokines and growth factors produced by tumor cells and immune cells.^32^ Secondly, the decrease in plasma ALB concentration reflects the poor nutritional condition of patients with cancers, which may be related to the chemotherapy resistance of patients.^33^ Further, the low concentration of serum ALB is related to the poor survival time of patients with various cancers,^32^ as well as for ovarian cancer.^34^ However, these studies have not clarified how low plasma levels of ALB could lead to the poor prognosis of patients with cancers, and our research may provide some new supports for these conclusions. In turn, these conclusions also support our findings. Moreover, the samples in our study were collected from patients with new‐onset HGSOC, which may provide a certain research basis for the further understanding of the role of ALB in the occurrence of HGSOC. APOB is the main apolipoprotein of chylomicrons and low‐density lipoproteins (LDL) and is the ligand for the LDL receptor,^35^ and the low or absent levels of APOB in plasma usually lead to familial hypobetalipoproteinemia and abetalipoproteinemia.^36^ Interestingly, increasing studies uncover that loss‐of‐function mutations of *APOB* frequently occur in multiple cancers including melanoma, liver cancer, stomach, esophageal, head and neck, uterine, and lung cancers. For liver cancer, Lee et al.^37^, ^38^ find that loss or inactivation of APOB in hepatocellular carcinoma is significantly associated with poor survival of HCC patients, whereas another group finds that elevated APOB predicts poor prognosis after surgery in patients with hepatocellular carcinoma,^39^ indicating that there is no consensus on the role of APOB in predicting the prognosis of hepatocellular carcinoma. Meanwhile, APOB also might be associated with the immune cell infiltration in cholangiocarcinoma.^40^ SERPINA1 is a serine protease inhibitor belonging to the serpin surperfamily, elevated level of which is related to the invasive potential of gastric, lung and colorectal adenocarcinoma,^41^, ^42^, ^43^ On the contrary, other studies have proved that SERPINA1 is significantly downregulated in a variety of cancers, and patients with high expression of SERPINA1 have a longer overall survival than patients with low expression, showing an anti‐cancer effect.^44^, ^45^, ^46^ The above completely opposite effects of APOB and SERPINA1 on predicting prognosis of tumors indicate the complexity of tumorigenesis and tumor development. But for HGSOC, there are currently no reports indicating an association between APOB or SERPINA1 and the tumorigenesis or prognosis of HGSOC, and our study provides first‐hand information for subsequent researches in this field.

The limitation of this study is that our results were left without verification due to the lack of other independent proteomic data. Secondly, due to the limitation of mass spectrometry technology, the relative abundance of most proteins is missing, and in order to ensure the reliability of the results, these proteins are not included into our analysis, which may cause bias to our conclusion. Furthermore, these findings need to be confirmed by further clinical practices in the future.

In summary, the HGSOC‐associated module was revealed through WGCNA. ALB, APOB and SERPINA1 were identified as the prognostic biomarkers for HGSOC, the protein levels of which were positively correlated with the survival time of patients with HGSOC.

## CONFLICT OF INTEREST

The authors declared that they have no potential conflicts of interest.

## Supporting information

Supplementary Material

Supplementary Material

Supplementary Material

Supplementary Material

## Data Availability

The data that support the findings of this study are available in CPTAC Data Portal at https://cptac‐data‐portal.georgetown.edu/studies/filters/primary_site:Ovary.
