# Supplementary material for: Integrated weighted gene co‐expression network analysis reveals biomarkers associated with prognosis of high‐grade serous ovarian cancer
Source: J Clin Lab Anal. 2022 Jan 8;36(2):e24165. doi: 10.1002/jcla.24165 (PMC8841170; doi:10.1002/jcla.24165)
Supplement: Supplementary file 1 — Supplementary Material [file JCLA-36-e24165-s002.pdf]

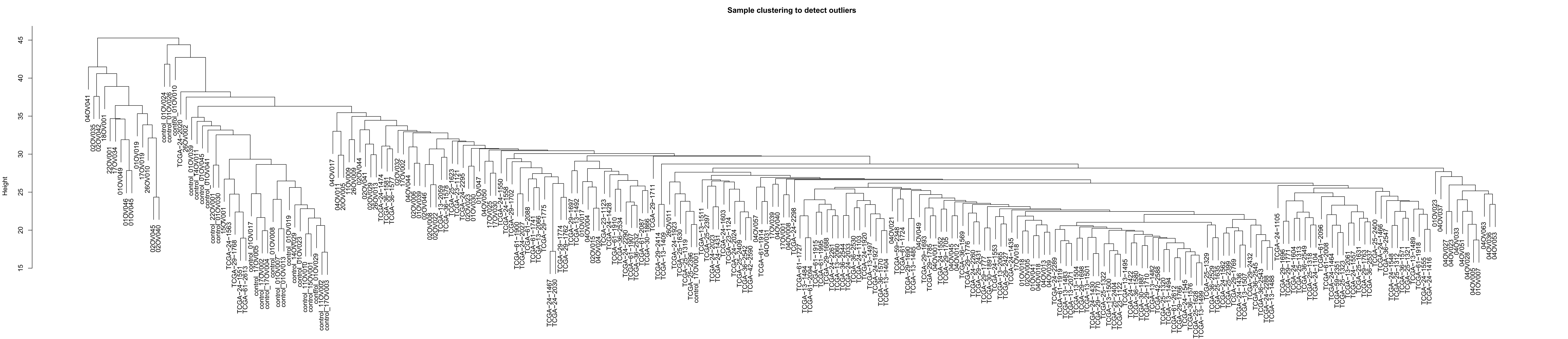
Sample clustering to detect outliers
Height
15
20
25
30
35
40
45
